# Individual longitudinal compliance to neglected tropical disease mass drug administration programmes, a systematic review

**DOI:** 10.1371/journal.pntd.0010853

**Published:** 2023-07-17

**Authors:** Rosie Maddren, Anna Phillips, Santiago Rayment Gomez, Kathryn Forbes, Benjamin S. Collyer, Klodeta Kura, Roy Anderson

**Affiliations:** 1 Department of Infectious Disease Epidemiology, Imperial College London, Saint Mary’s Campus, Norfolk Place, London, United Kingdom; 2 FHI 360, Durham, North Carolina, United States of America; 3 London Centre for Neglected Tropical Disease Research (LCNTDR), Imperial College London, Saint Mary’s Campus, Norfolk Place, London, United Kingdom; RTI International, UNITED STATES

## Abstract

Repeated distribution of preventative chemotherapy (PC) by mass drug administration forms the mainstay of transmission control for five of the 20 recognised neglected tropical diseases (NTDs); soil-transmitted helminths, schistosomiasis, lymphatic filariasis, onchocerciasis and trachoma. The efficiency of such programmes is reliant upon participants swallowing the offered treatment consistently at each round. This is measured by compliance, defined as the proportion of eligible participants swallowing treatment. Individually linked longitudinal compliance data is important for assessing the potential impact of MDA-based control programmes, yet this accurate monitoring is rarely implemented in those for NTDs. Longitudinal compliance data reported by control programmes globally for the five (PC)-NTDs since 2016 is examined, focusing on key associations of compliance with age and gender. PubMed and Web of Science was searched in January 2022 for articles written in English and Spanish, and the subsequent extraction adhered to PRISMA guidelines. Study title screening was aided by Rayyan, a machine learning software package. Studies were considered for inclusion if primary compliance data was recorded for more than one time point, in a population larger than 100 participants. All data analysis was conducted in R. A total of 89 studies were identified containing compliance data, 57 were longitudinal studies, of which 25 reported individually linked data reported by varying methods. The association of increasing age with the degree of systematic treatment was commonly reported. The review is limited by the paucity of data published on this topic. The varying and overlapping terminologies used to describe coverage (receiving treatment) and compliance (swallowing treatment) is reviewed. Consequently, it is recommended that WHO considers clearly defining the terms for coverage, compliance, and longitudinal compliance which are currently contradictory across their NTD treatment guidelines. This review is registered with PROSPERO (number: CRD42022301991).

## Author summary

Neglected tropical diseases (NTDs) are estimated to affect 1.74 billion people globally, often those in low socio-economic communities in tropical and sub-tropical regions. Five NTDs can be effectively treated using repeated administration of preventative chemotherapy (PC), delivered to communities with endemic infection. This is described as mass-drug administration (MDA). Repeated treatment is necessary due to re-infection of treated people by those untreated in these endemic communities, and the absence of strong acquired immunity in the human host to many of the NTDs despite repeated exposure to infection. Increasing the number of people treated at each round is critical to increase the number of parasite-free individuals. As a result, this will reduce the amount of re-infection to the community and therefore increases the chance of reaching the goal of transmission elimination. Currently, the measurement of MDA success is focused upon coverage defined as the *receiving* of treatment. However, not everyone who receives treatment *swallows* the treatment, which defines compliance. Reviewing patterns of compliance over time in the population targeted for treatment, such as the proportion of the population which always takes treatment, or conversely never takes treatment, will help predict the impact of MDA on disease prevalence and intensity. This review summarises the results of recent studies recording compliance data for the five NTDs controllable by MDA, soil-transmitted helminths, schistosomiasis, lymphatic filariasis, onchocerciasis and trachoma.

## Introduction

There are an estimated 1.74 billion people requiring interventions against the neglected tropical diseases (NTDs) [1]. This has reduced by 600 million since 2010, yet considerable progress is still required to further reduce the global burden of disease caused by these species [1]. The recently published WHO 2030 roadmap for NTD control clearly demonstrates the link between socioeconomic status and NTD burden, highlighting the inverse relationship between increasing GDP per capita with decreasing NTD burden [1]. NTDs impart physical and financial sequalae, and infection status (often resultant from poor hygiene or living conditions due to poverty) can further reduce an individual’s potential to earn, creating a downward spiral in the well-being of an infected individual. As NTDs disproportionately affect individuals of low socioeconomic status, self-funded care is not feasible for most affected, making global control of parasites predominantly reliant upon drug donations from pharmaceutical companies and operational support of treatment programmes from international philanthropic organisations.

There are 20 NTDs recognised by WHO, five of which are responsible for the majority of the global NTD burden. These are lymphatic filariasis (LF), onchocerciasis (caused by *Onchocerca volvulus* (OV)), schistosomiasis (SCH), soil-transmitted helminths (STH), and trachoma. All these parasitic infections can similarly be treated effectively by preventative chemotherapy (PC), hence the title (PC)-NTDs, albeit Trachoma control implementing this as part of the wider ‘SAFE’ strategy [2]. Entire endemic communities or at-risk subgroups receive mass drug administration (MDA) to treat the parasitic infection and control transmission, substantially reducing morbidity. Prior infection or treatment does not confer strong acquired immunity to any of these five parasites, thus repeated treatments are necessary due to re-infection of treated individuals by those who were not treated. The frequency and time interval between these repeated treatments is dependent upon prevalence levels and the life expectancy of the parasite in the human host [3,4]. Compliance (receiving and swallowing of drugs) across the targeted eligible population rarely, if ever, reaches 100%, further adding to the requirement for repeated treatments to lower parasite prevalence [4]. Following decades of MDA, parasite prevalence levels have dropped markedly in the majority of NTD endemic communities. Currently, elimination of parasites as a public health problem has been achieved by 17 countries for LF and 10 countries for trachoma [1]. As such, a number of countries and regions can realistically consider elimination of transmission, as is the current goal for OV, or elimination as a public health problem (targeted for LF, STH, SCH and trachoma) [1].

To avoid persistent reservoirs of infection, MDA delivery must achieve high compliance (swallowing of drugs) at each round to optimise programmatic success. Coverage targets are provided by WHO, however currently no clear definitions or targeted goals exist for compliance to any of the five PC-NTDs. This is of concern in settings aiming for transmission elimination, as the coverage-compliance gap (difference between those receiving treatment and those swallowing treatment), can sometimes reach up to 20% [5]. Control programmes misconstruing coverage and compliance can therefore grossly underestimate the treated population. The recently published WHO 2030 NTD roadmap [1] would benefit future (PC)-NTD control progress by defining distinct coverage and compliance targets for each species.

Donor fatigue and economic pressures currently threaten the frequency and scale of NTD control programmes, reversing the successes made to date. This makes the improved monitoring and subsequent enhancement of compliance behaviour particularly important at present. However, even if compliance reached satisfactory levels for transmission elimination, it should be noted that drug efficacy for each NTD species is not always 100%, further complicating the interpretation of coverage and compliance upon control impact. Albendazole, the most widely used drug to treat STH infection, has a range of efficacies for different parasites, from *Ascaris lumbricoides* (91.4%) to *Trichuris trichiura* (50.0%) [6]. This demonstrates the requirement of monitoring compliance by age and gender, enabling stratified compliance data to be reviewed against the known age-dependent distribution of prevalence, which varies between parasitic species. Furthermore, the armoury of NTD control includes many intervention methods, one of which is MDA. Implementation of improved water, sanitation and hygiene (WaSH) infrastructure, often conducted alongside MDA programmes, will reduce the environmental contamination of infectious material, further reducing the transmission potential of a community [7]. This impact of WaSH is beyond the scope of this review, but importance is nonetheless noted.

**Fig 1:**
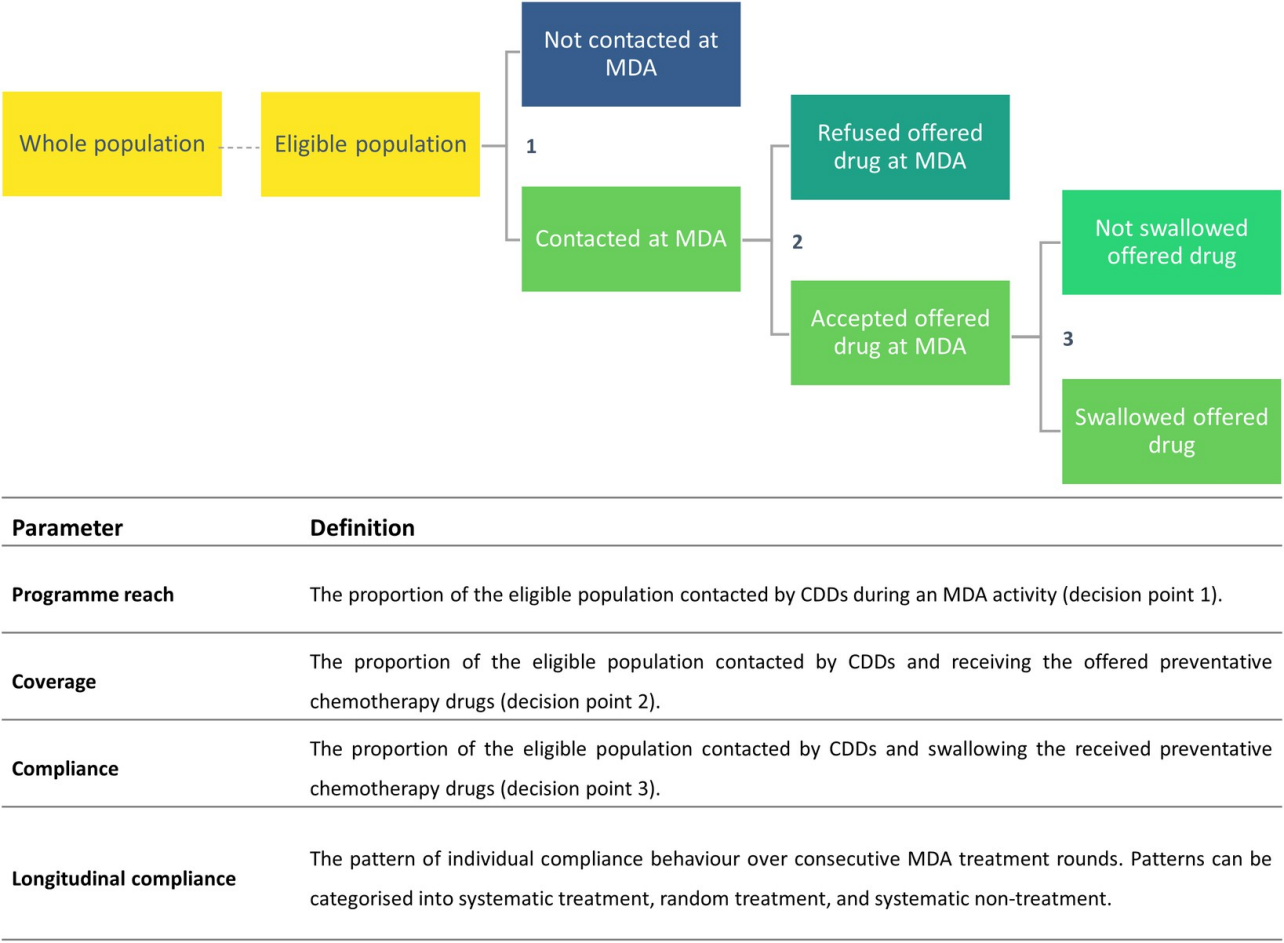
Decision tree of MDA treatment behaviour, showing the three behavioural decision points of MDA delivery and their definitions, according to these previously defined parameters by Shuford et al. (2016) [8]. Abbreviations: CDD–community drug distributor.

The key epidemiological parameters often used to monitor and evaluate the success of control programmes can be divided into three key decision points of individual MDA behaviour as recorded in **Fig 1**, defining the interaction between a community drug distributor (CDD) and participant [8]. Similar decision trees have been published to help clearly define these parameters [9,10]. In this review (and in keeping with the terminology previously employed [8,11]), the term *coverage* is used to refer to the proportion of the eligible population contacted by the CDD, who received the offered drugs. *Compliance* is often confused or conflated with coverage. In this review it is defined as the proportion of the eligible population who were contacted by CDDs and swallowed the received drugs. The importance of the distinction between these two epidemiological parameters in NTD research was first highlighted by Babu and Babu (2014) in the context of reaching LF transmission break in India [5], but its importance has been identified in other disease areas since 1982 [12].

Rather than evaluating each MDA treatment round as a unique activity, MDA programmes must also focus on individual longitudinal patterns of compliance to treatment over time. Ideally, this would be measured by directly observed treatment (DOT), avoiding any recall or desirability bias created from participant’s recollecting past treatment. The term “longitudinal compliance” is defined in this review as the monitoring of the same individual’s treatment behaviour across multiple time points. Longitudinal studies of compliance enable the identification of individuals who systematically do or do not swallow drugs over consecutive rounds of MDA. We refer to these groups as *systematically treated* or *systematically non-treated*, respectively [13,14]. The terminology used to describe systematic non-treatment has evolved in recent years, to reflect the agency held by the participant and their behaviour. Systematic non-treatment, or the ‘never treated’ population has previously been referred to as non-adherence, or systematic non-compliance. Pockets of ‘never treated’ individuals are resultant from patterns of systematic treatment, these individuals act as an infection reservoir for re-infection, making the converse, random patterns of compliance, across these repeated rounds preferential [15]. The importance this systematically non-treated proportion of the population holds in the context of transmission elimination is dependent upon its magnitude and the intensity of transmission in a defined locality as measured by the basic reproductive number, R_0_, of the parasite. As such, if there is a high proportion of non-treatment within a population, this is more problematic for effective control for parasites with a higher R_0_ compared to those with a low R_0_ [12,16].

The description of demographic associations (age and gender) of systematically non-treated populations over contiguous rounds of MDA has not been well-defined in the literature to date [8,17]. This review focuses on published studies of longitudinal compliance in defined populations, and the linkage of treatment behaviour patterns with age and gender. A systematic review of the available data published for the five (PC)-NTDs until 2022 was conducted, updating the previous review by Shuford et al. (2016) [8].

## Methods

The systematic review was conducted in line with the PRISMA guidelines [18] (**S1 PRISMA Checklist**) and is registered with PROSPERO (Registration number: CRD42022301991). Studies detailing systematic non-treatment to MDA for LF, OV, SCH, STH, and trachoma were searched using a Boolean strategy across PubMed and Web of Science databases in January 2022. The search strategy used and resulting PRISMA workflow are detailed in **S1 Data and S1 Note**. This is an update of the review previously conducted by Shuford *et al*. (2016) [8] which was considered to be completed comprehensively. As such, only studies published from 2016 up to 2022 written in English and Spanish were considered for inclusion. Minimal inclusion criteria were used due to the paucity of the relevant studies identified. These were: 1) primary compliance data reported from community or school-based control programmes targeting LF, OV, SCH, STH, and trachoma infections, 2) at least two defined time points (longitudinal data), and 3) sufficiently large population sample (>100 participants). Where reported, reasons given for non-treatment were collected for subsequent meta-analyses. All data collected was thoroughly checked to be defined as compliance (swallowing of drugs). Irrespective of the term employed in papers reviewed for extraction, data reported using the compliance as defined in this review was extracted. Definitions used in the reviewed publications to incorrectly label compliance included “programme / epidemiological coverage”, “uptake”, and “adherence”.

Two search engines (Web of Science, PubMed) identified 3,649 studies, which were amalgamated in Excel and de-duplicated. In total, 2,529 unique studies were then transferred to Rayyan, an online software package using natural language processing and machine learning for data evaluation [19]. Using this software, whilst blinded, authors conducted title and abstract screening, categorising paper themes as quantitative (n = 164) for inclusion and excluding those categorised as qualitative (n = 40), modelling (n = 45), and not relevant (n = 2280). Despite the exclusion of qualitative studies from this analysis, some themes presented in these studies were used to help interpret the data extracted in this review. Overall, 164 quantitative studies were read in full and assessed for eligibility for data extraction. The references in these papers were screened to capture any further relevant data, generating one additional article for inclusion. From the 165 total studies identified, 6 studies were not publicly accessible, and a further 70 were excluded due to containing coverage data (n = 30), or irrelevant data (n = 40). The remaining 89 studies (datasets) were confirmed to report compliance data and were therefore eligible for data extraction (**S1 Note**). Data extracted from the 89 studies included the study type, sample type (including age and gender stratifications where available), number of studied participants, length of study, geographic identifiers, drugs provided, definitions of numerator and denominator for reported parameters, terminology used to describe parameters, and reasons for not complying with treatment. Multiple datapoints were extracted from studies when detailed stratifications such as age, regions and individual parasite species satisfied the extraction criteria. Coverage was extracted when reported alongside compliance for comparative analyses. Due to the paucity of papers containing individual longitudinal compliance data, compliance papers were categorised in a tiered format as containing; individual compliance data (recorded by CDD), individual compliance data (self-reported), repeated cross-sectional compliance data and cross-sectional compliance. Data was analysed in R (4.2.2 (2022-10-31) "Innocent and Trusting") [20] and all figures were generated using the R package ggplot2 [21] unless specified.

## Results

A total of 165 studies met the inclusion criteria for data extraction (see **S2 Data**). Of these, 46 papers were rejected from the full-text review as irrelevant. Reasons for rejection included data reported in an ineligible format e.g. averages (n = 25), the reporting of only secondary data (n = 8), inability to access full-text data (n = 6), targeted treatment studies (n = 3), and reporting on sample sizes smaller than 100 (n = 2). There were 30 studies identified as reporting cross-sectional (n = 7) and longitudinal (n = 23) coverage data, not compliance data, as the abstract and title would have suggested. Removing the irrelevant studies, including studies reporting coverage data, left a total of 89 studies to be considered in the analyses. An overview of these studies is provided in **Table 1**.

**Table 1:** Summary overview of the 89 studies included in the analysis split by the epidemiological classification of cross-sectional or longitudinal data. The full data extraction of the 165 studies is provided in **S2 Data.** Frequency and percentage of studies falling into each category is shown as “n (%)”. Note for ‘Sample Population’, each stratification totals the number of studies, whilst the remaining rows of the table represents a single stratum. Abbreviations: LF–lymphatic filariasis, N–number of studies, OV–onchocerciasis, preSAC–preschool-aged children, SAC–school-aged children, SCH–schistosomiasis, STH–soil-transmitted helminths.

		Cross-sectional (n (%)) (N = 32)	Longitudinal (n (%)) (N = 57)
**Sample Population**	preSAC	0 (0)	0 (0)
	SAC	8 (25)	6 (11)
	Adults	2 (6)	9 (16)
	Community	22 (69)	38 (67)
	Other	0 (0)	2 (4)
**Stratification**	Age	12 (38)	13 (23)
	Gender	13 (41)	14 (25)
	Region	18 (56)	22 (39)
	None	8 (25)	35 (44)
**Species**	STH	9 (28)	9 (16)
	SCH	11 (34)	6 (11)
	OV	4 (12)	18 (32)
	LF	16 (50)	29 (51)
	Trachoma	3 (9)	3 (5)

### Stratification of compliance data

Across the 89 included studies, those stratifying compliance data by location were frequent, employed by 56% and 39% of the cross-sectional and longitudinal studies, respectively. Age and gender stratifications were reported by a smaller proportion of studies. Whole communities most frequently formed the sample population, employed by 69% and 67% of cross-sectional and longitudinal studies, respectively. Sample populations categorised as ‘other’ used non-standard age breakdowns such as 0–14 year-olds, and 2–14 year-olds. The (arithmetic) mean sample size of cross-sectional studies ($\bar{x}$ = 4965) was considerably smaller than that of longitudinal studies ($\bar{x}$ = 1,257,386). Five of the longitudinal studies reported compliance in populations ranging from 1–400 million, reported from national control programmes in Myanmar [22], China [23], Sierra Leone [24,25], and the Dominican Republic [26].

Within the 89 studies included in this analysis, 78 studies reported MDA data for a single parasite species. An additional 30 datapoints were generated by 11 studies reporting compliance data for two (n = 10), three (n = 12), and four (n = 8) species together, totalling 108 species-specific datapoints for analysis. Overall, 45, 22, 18, 17 and 6 studies reported compliance data for LF, OV, STH, SCH, and trachoma. Studies focusing solely upon LF (n = 37) were most numerate and were found to be most consistently published between 2016 and 2022. Fewer studies were published focused solely on OV (n = 17), STH (n = 9), SCH (n = 9) and Trachoma (n = 6). The most frequently reported species combination was STH and SCH (n = 3). Trachoma studies were least common and were reported only as a single species, never in combination with other parasites. This is presumably due to different control methods, as Azithromycin is administered in accordance with the SAFE strategy for Trachoma control [27]. No trachoma compliance studies have been published since 2019.

**Fig 2:**
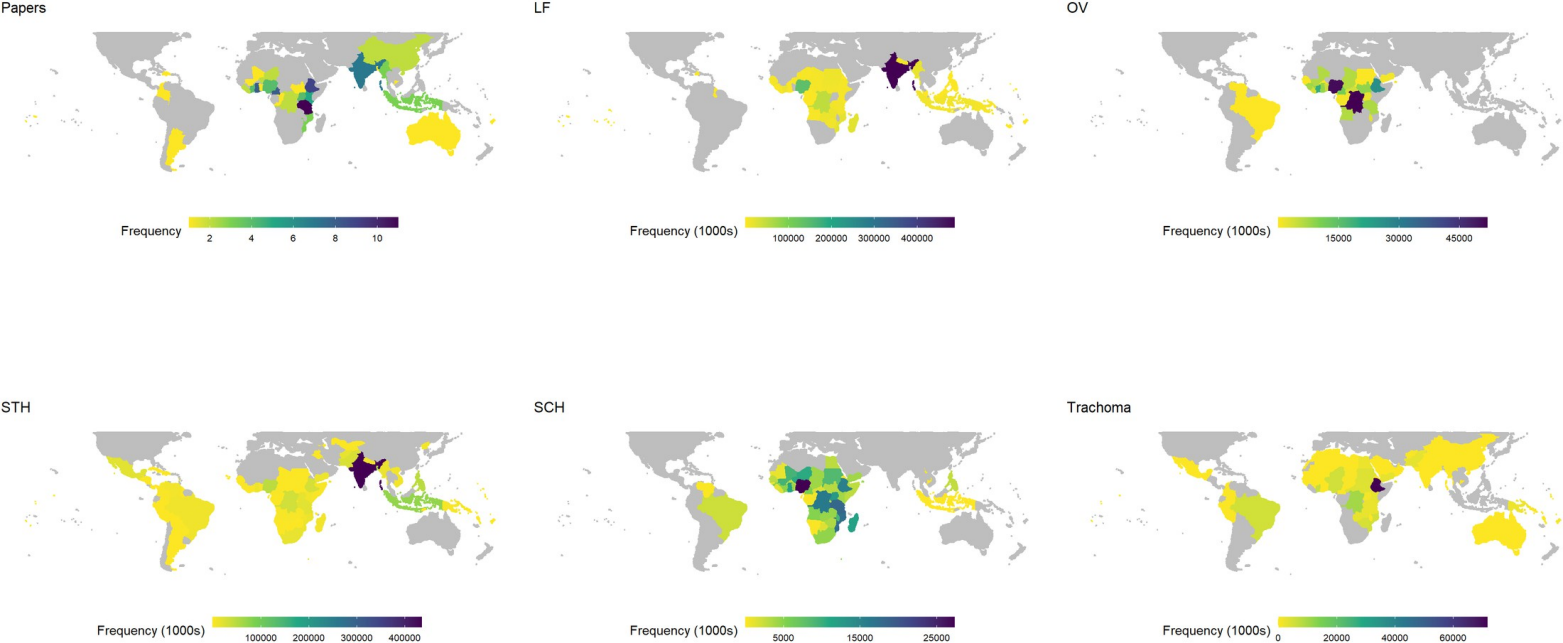
Choropleth map of studies published globally from 2016 to 2022 (Papers). Countries where no papers were published are shown in grey. Four studies are represented as 11 datapoints where studies reported data for more than one country, totalling 96 datapoints from 89 studies. (**LF, OV, STH, SCH, Trachoma**) Maps showing total population of each country requiring treatment for each of the five (PC)-NTDs. Note: STH map shows total preSAC and SAC population only requiring treatment. All maps have been focused to latitude less than 50 degrees. Data taken from WHO global observatory PCT databank [28]. Abbreviations: LF–lymphatic filariasis, OV–Onchocerciasis, SCH–schistosomiasis, STH–soil-transmitted helminths. Map data acquired from the open source tidyverse package using the map_data() function [29].

From the 89 studies, an additional 11 geographical datapoints were generated from four studies reporting data from multiple countries, providing a total of 96 geographic datapoints. Globally, studies reporting compliance from Tanzania (n = 11) and Ethiopia (n = 9) were most prevalent, as depicted in **Fig 2**. The total population requiring treatment globally as reported by the WHO (PC)-NTD databank [28] highlight the geographical gaps in MDA compliance research, specifically there was little data from southern African countries for SCH and STH, and northern African countries and northern South America countries for Trachoma research.

### Incongruent parameter definitions

**Fig 3:**
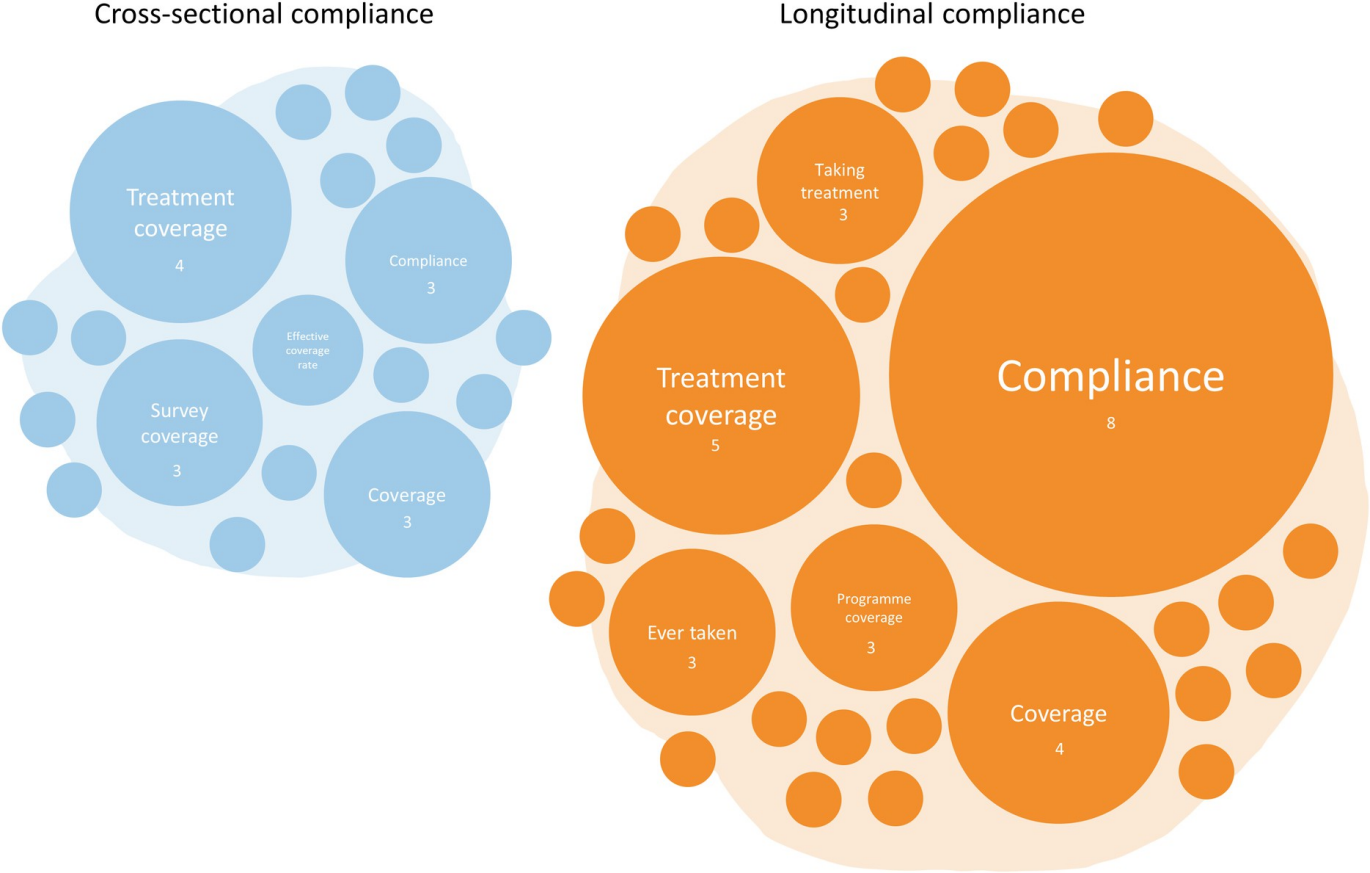
The variety of parameters used to describe cross-sectional and longitudinal compliance. The display shows the most frequently used parameters both correctly and incorrectly used to describe compliance. The frequency of parameter definition is proportional to circle size. Compliance is used most frequently to describe longitudinal compliance (n = 8), whereas treatment coverage was most frequently used to describe cross-sectional compliance (n = 4). The number of circles represent the number of parameters employed to define compliance. Figure hand-drawn by authors.

The reported numerator, denominator, and definitional calculations were carefully analysed for all reviewed studies. Where provided, these were often defined incompletely. When parameters reported did not match that used in this review, standardised parameters were assigned to the data. Often, compliance was misconstrued as coverage. The variety of parameters employed by the reviewed publications to describe cross-sectional and longitudinal compliance as defined in this review is recorded in **Fig 3**.

From the numerators and denominators provided, just 26 (29%) studies could be confidently identified as containing either cross-sectional or longitudinal compliance data (the proportion of the eligible population who were contacted by CDDs and swallowed the offered drugs). Compliance data defining either the correct numerator or denominator for compliance, not both, were categorised as ‘assumed’ (n = 12). Due to the wide variety of definitions used for the same parameter across studies (see **Fig 3**), publications that did not define either numerator or denominator for the parameter given were classified as undefined (n = 5). These parameters were “epidemiological coverage”, “MDA participation”, “treatment coverage”, “MDA coverage” and “compliance”.

The parameter “treatment coverage” (n = 4) was used most frequently to describe cross-sectional compliance, whereas longitudinal compliance was most frequently, correctly referred to as “compliance” (n = 8). Compliance was only correctly used in three of the 32 cross-sectional studies where the numerator and denominator were clearly defined [10,30,31].

### Categorisation of compliance studies

Due to the paucity of the available literature detailing individual longitudinal compliance recorded by DOT, a hierarchy of studies was created, encompassing both longitudinal and cross-sectional studies as detailed in **Table 2**. The 57 (64%) studies containing longitudinal data were categorised as reporting individual data (recorded by data collectors (n = 3) or self-reported (n = 22)), repeated cross-sectional data (n = 32), or cross-sectional data (n = 32) (**Table 2**).

**Table 2:** Data type and definition used to classify 57 compliance papers included in the analysis. Longitudinal compliance data was reported by both longitudinal studies (cohort, randomised controlled trials) and cross-sectional studies. Abbreviations: CDD–community drug distributor, DOT–directly observed treatment.

Compliance Data Type	Study Type	Description
**Individual–annually surveyed (n = 3)**	Cohort, randomised controlled trial	Longitudinal, individually linked, compliance data across >2 time points. Recorded DOT during annual surveys either by CDD, or self-reported by participant. Focus: individual level.
**Individual–self-reported (time-specific or overall) summary (n = 22)**	Cross-sectional	Longitudinal compliance data over defined/undefined time-period. Self-reported compliance by an individual at one time-point, looking back in time e.g., self-reported compliance to 5 available MDA rounds. Focus: individual level.
**Repeated cross-sectional (n = 32)**	Cross-sectional	Compliance data at each round of MDA for a set cohort (e.g., village or district), repeatedly surveyed over several time points population repeatedly sampled. Individuals not consistently present across all time points are not removed. Focus: population level.
**Cross-sectional (n = 32)**	Cross-sectional	Compliance data for a single round of MDA. Focus: population level.

Of the 25 individual compliance studies, the most frequently employed method of measuring this was to ask participants if they had ‘ever’ been treated (n = 15), either over a defined time period of 5 years, 6 years, 7 years, 12 years, or the participant’s lifetime. Annual and biannual studies were found at equal frequencies in the review (n = 5). Biannual studies tended to follow fewer rounds of MDA (range: 2–10 rounds), compared to annual studies (range: 4–12 rounds). Studies reporting individual longitudinal compliance increased slightly over the time period 2016 to 2022, peaking with four studies reported in 2021 compared to zero in 2016. Similarly, the number of cross-sectional compliance studies published also slightly increased, with seven studies reported in 2021.

A total of 32 studies (36%) reported cross-sectional (single time-point) compliance data. As these studies report compliance for just one time point, these studies are disregarded from any further longitudinal analysis in this review. A further 32 studies reported repeated cross-sectional compliance data over time periods ranging from a year of biannual treatment to 26 years of annual treatment. As these datasets follow compliance at a population level, these studies are not categorised as containing individual longitudinal compliance data, and are also disregarded from any further analysis.

### Individual longitudinal compliance data

A sample of the extracted columns from the review is shown below for the 12 of the 25 individual compliance studies containing both annually surveyed and self-reported individual compliance data in **Table 3**. A single study was identified as containing annual longitudinal compliance data recorded by CDDs at point of delivery, referring to the parameter as “systematic treatment” [17]. The study reported on the Kenyan project TUMIKIA, where treatment behaviour of 36,000 individuals’ for four rounds of biannual MDA data for STH was digitised, linking individual treatment behaviour longitudinally via house hold occupancy [17]. In a further two studies, individuals self-reported their compliance to the previously administered round of MDA, providing data for each round of LF MDA in the Democratic Republic of the Congo and the Republic of Congo [32], and STH MDA in China [33]. These studies were categorised as ‘assumed’ longitudinal compliance data, due to incomplete definitions of parameter numerators and denominators, referring to longitudinal compliance as either “treatment adherence” [32], or “taking treatment” [33].

**Table 3:** A selection of the extracted columns for the 12 studies satisfying the criteria for individual-level data–both annually surveyed by CDD or self-reported by participants (grey shading), and self-reported during a cross-sectional analysis. Available stratifications of compliance data are shown from the possible categories of age, gender, or location. The species targeted for control by MDA, and the drug offered for which compliance to is measured, is also given. The country, and the type and number of participants is also provided. The terminology used to describe compliance is shown, along with the ‘true’ definition according to this review given the numerator and denominator recorded (where given). Where there are incomplete numerator or denominators provided, compliance can only be assumed given the large heterogeneity of calculations used for the parameter. Abbreviations: ALB–Albendazole, IVM–Ivermectin, LF–lymphatic filariasis, OV–onchocerciasis, SAC–school-aged children, SCH–schistosomiasis, STH–soil-transmitted helminths.

Author	Compliance type	Stratification	Species	Drug	Country	N	Sample Type	Terminology Used	Standardised Definition	Numerator	Denominator
Oswald et al. (2020) [17]	Individual: 4 biannual rounds over 2 years—recorded by CDDs.	Age, gender	STH	ALB	Kenya	36327	whole community	Systematic treatment	Longitudinal compliance	Eligible participants swallowing	Eligible population
Campillo et al. (2021) [32]	Individual: 8 biannual rounds. Self-reported compliance to previous round at each subsequent round.	-	LF	ALB	Republic of Congo, Democratic Republic of the Congo	2658	whole community	Adherence	Assumed longitudinal compliance	Not given	Not given
Liu et al. (2017) [33]	Individual: 2 biannual rounds. Self-reported at each time point.	-	STH	ALB	China	10000	SAC	Taking treatment	Assumed longitudinal compliance	Not given	Eligible population
Tilahun et al. (2018) [34]	Individual—as previously taken: 6 annual rounds. Self-reported compliance at each round.	Age, gender	Trachoma	AZT	Ethiopia	5826	whole community	Coverage	Longitudinal compliance	Eligible participants swallowing	Eligible population
Osue (2017) [35]	Individual—as previously taken: 10 annual rounds. Self-reported compliance to each round.	Location	OV	IVM	Nigeria	438	adults	Taking treatment	Assumed longitudinal compliance	Not given	Not given
Wanji et al. (2018) [36]	Individual—as ever taken: Self-reported compliance to 1–11+ annual rounds.	Age, gender, location	OV	IVM	Cameroon	3684	whole community	Ever treated / MDA adherence	Assumed longitudinal compliance	Not given	Eligible population
Manyeh et al. (2020) [37]	Individual—as ever taken: Self-reported. No limit given.	Location	LF	IVM, ALB	Ghana	446	adults	Compliance	Longitudinal compliance	Eligible participants swallowing	Eligible population
Minetti et al. (2019) [38]	Individual—as ever taken: Self-reported. (Maximum median rounds– 8)	Location	LF	-	Ghana	924	adults	Ever treated / MDA adherence	Assumed longitudinal compliance	Not given	Not given
Manyeh et al. (2021) [39]	Individual—as ever taken: Self-reported. Pre- and post-intervention.	-	LF	-	Ghana	446	whole community	Ever taken	Assumed compliance	Not given	Not given
Aza’ah et al. (2020) [40]	Individual—as ever taken: Self-reported, over 5 years.	Age, gender	OV	IVM	Cameroon	603	whole community	Compliance	Longitudinal compliance	Eligible participants swallowing	Eligible population
Forrer et al. (2021) [41]	Individual—as ever taken: Categorised into never, 0–50%, 50–75%, 75%+ compliance to possible rounds.	Age	OV	IVM	Cameroon	19915	whole community	Adherence	Assumed longitudinal compliance	Not given	Eligible population
Kifle et al. (2021) [42]	Individual—as ever taken: 10 biannual rounds. Self-reported.	Age, gender, location	OV	IVM	Ethiopia	553	adults	Compliance	Assumed longitudinal compliance	Not given	Eligible population

Longitudinal compliance studies reported over a time-specific period were more common (n = 22). Two longitudinal studies asked respondents to recall their previous treatment behaviour at each round, over six annual rounds in Ethiopia for trachoma [34], and over 10 annual rounds in Nigeria for OV [35]. Three cross-sectional studies asked participants to recall their treatment behaviour during MDA for OV over a specific time period, either five annual rounds [40] and 11+ annual rounds [36] in Cameroon, or 10 biannual rounds in Ethiopia [42]. Two of these studies referred to compliance in line with the definitions considered for this review [40,42]. A further two cross-sectional studies asked participants to recall ever being treated during MDA for LF in Ghana [37,38]. One study reported longitudinal compliance as a percentage of the possible rounds per participant based upon eligibility, breaking compliance into ‘never’, or complying with “≤50%”, “50–75%”, “≥75%” of possible rounds of MDA against OV in Cameroon [41]. One study reported the change in compliance before and after an interventional effort to increase MDA uptake for LF control in Ghana [39].

A combination of cross-sectional and longitudinal compliance was reported by 13 studies, whereby compliance to a defined [43–50] or undefined [51–55] number of rounds were reported, alongside compliance at the latest round of MDA. Four of these studies defined compliance in line with the definitions considered for this review [45,51–53]. Setting a high reporting standard, Osei *et al*. (2022) [53], Oswald *et al*. (2016) [17] and Krentel *et al*. (2016) [55] provided comprehensive demographic and descriptive factors and associations with receiving, and swallowing MDA through bivariate and multivariate logistic regression analysis. This high level of descriptive detail for coverage and compliance was rarely observed in the published literature. The research landscape of MDA reporting and its impact on infection levels would greatly benefit from more studies following these detailed study designs.

### Compliance versus coverage

**Fig 4:**
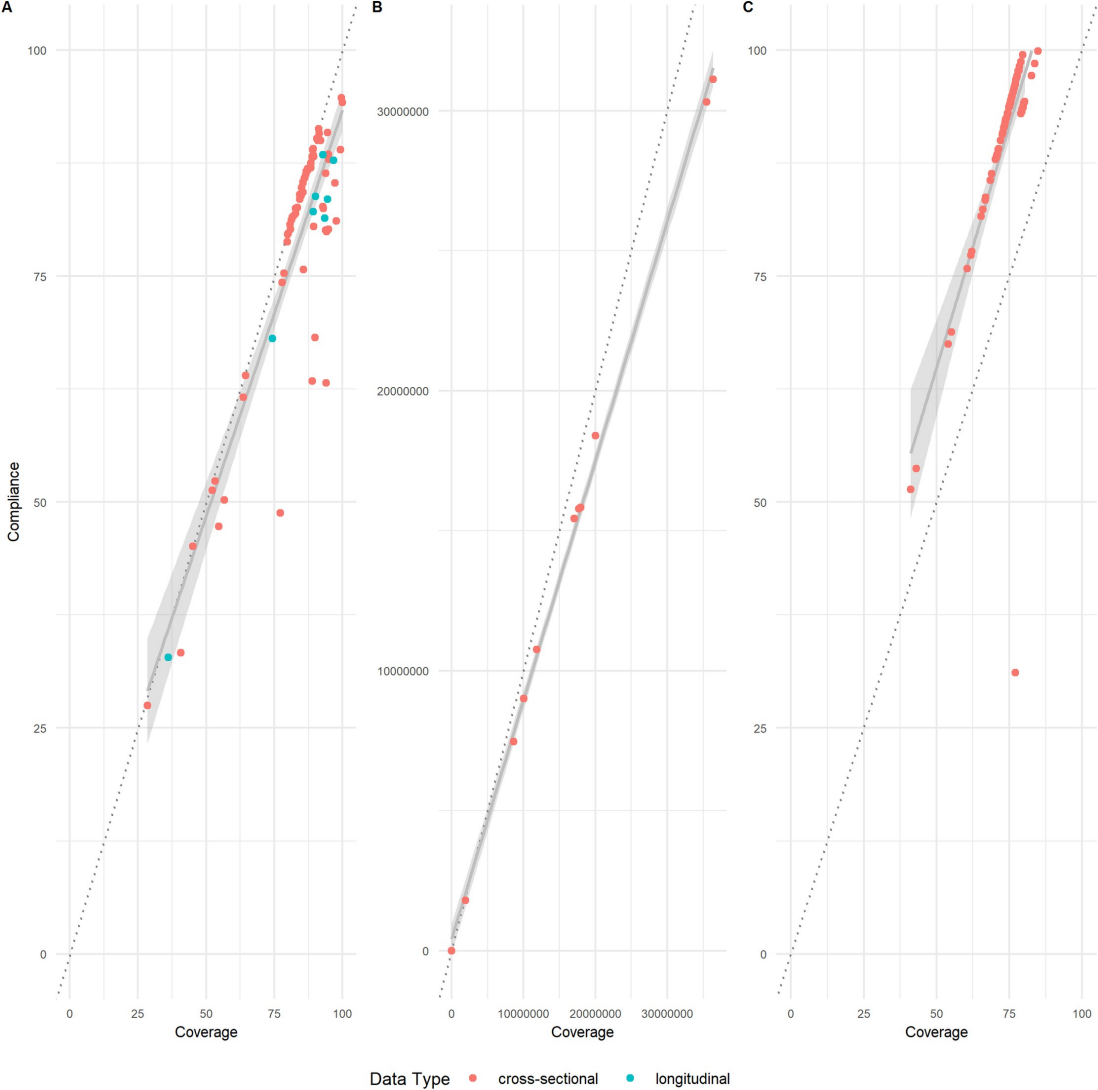
The difference between coverage and compliance figures reported by 21 studies as either proportions (**A**), raw data (**B**) or other parameters (**C**). Datapoints are coloured by the study type, either cross-sectional (13 studies, 69 datapoints) or longitudinal (8 studies, 104 datapoints). An average trendline of the reported data is shown in solid grey. The shaded line represents the conditional mean function using a linear model calculated by geom_smooth within the ggplot2 R package [21]. A guideline through intercept = 0 is shown to highlight the discrepancy between the two parameters.

From the 32 and 57 cross-sectional and longitudinal studies reporting compliance, 41% (n = 13) and 14% (n = 8) reported coverage data alongside compliance data. These 21 studies produced 173 datapoints of comparable coverage and compliance data for different demographic strata, namely age, gender, location, and programme year. Uniquely, Chami et al. (2017) [10] calculated the average marginal effects (AME) for coverage and compliance against a variety of factors. Most commonly, coverage was reported as a proportion of the eligible population (studies = 16, datapoints = 77), but studies also reported the parameter as a raw number (studies = 2, datapoints = 11) or as different named parameters such as epidemiological, programme and geographic coverage (studies = 3, datapoints = 85). There was a mean average difference of 4.91% (min: 0%, max: 30.8%) between coverage and compliance figures reported as a proportions. Similarly, there was a 11.9% (min: 7.04%, max: 21.5%) mean average percentage change between the raw data figures reported. A larger discrepancy of 17.7% (min: 10.3%, max: 45.9%) was noted between epidemiological / programme coverage, and compliance / geographical coverage. The difference between the two parameters is shown in **Fig 4**.

### Stratification and analysis of compliance by demographic and behavioural factors

Stratifying the compliance data by demographic and behaviour factors provides information to highlight target groups for enhanced sensitisation and or mobilisation in MDA treatment. A total of 28 studies analysed key factors elucidating positive and negative associations influencing compliance in the studied population. Three main methods were employed for the analysis of compliance strata, mainly univariate, bivariate, and multivariate regression (presented as adjusted odds ratios (AOR)), Chi-squared analysis, and proportional breakdowns. Beyond the age, gender and location strata previously mentioned, further analyses were performed against enrolled/non-enrolled SAC, highest education level achieved of respondent, socioeconomic status, community type (rural/urban), marital status, occupation, ethnicity, religion, and length of stay in current village location.

Compliance increasing with age was noted by 12 studies. Specifically, compliance to treatment increasing with age was reported by Osei et al. (2022) [53] (AOR 4.23 (95% CI: 0.84–21.19), adults aged >58, compared to 18–27 year olds), and Chisha et al. (2020) [56] (AOR 1.45 (95% CI: 1.25–1.69), SAC aged 10–14, compared with to 5–9 year olds). Aza’ah et al (2020) [57] presented this phenomena via Chi-squared statistics, noting the proportion of age groups complying with MDA increased from 22.6% (95% CI: 16.3–30.4%) of 10–19 year olds, to 60.3% (95% CI: 53.1–67.1%) of participants aged ≥ 50 years (χ2 = 64.1, df = 3, P < 0.0001), as did Minetti et al. (2019) [38], who noted MDA participation increased with age (χ2 = 8.75, df = 1, p = 0.003). Mirroring this, Kifle and Nigatu (2021) [42] noted low compliance in young age groups, (AOR 0.68 (95% CI: 0.77–1.76) young adults aged 15–24 years, compared to >45 year olds). The variety of methods used to present age-related risk-factors with compliance prevents a subsequent meta-analysis to be conducted. Inconsistent conclusions were drawn for gender-related compliance behaviour. Statistical significance was noted for children enrolled in school, compared to those who were not (AOR = 20.90 (95% CI: 17.41–25.08) [56], AOR 2.29 (95% CI: 1.13–4.61) Asfaw et al. (2021) [58]).

Six studies noted an increased odds of compliance based upon previous compliance. This treatment behaviour can be described as systematic compliance, whereby an individual’s behaviour at any given round of MDA is dependent of behaviour at previous rounds. This phenomenon was described in different ways across the reviewed publications, varying between increased compliance based upon previous compliance (AOR 10.58 (95% CI: 5.78–19.38)) [53], willingness of future compliance by Tilahun and Fenta (2018) [34] (AOR 5.78 (95% CI:2.44–13.68)), and non-compliance related to previous non-compliance reported twice; (AOR 1.620 (95% CI: 1.051–2.497)) [38] and (AOR 9.18 (95% CI: 2.43–34.65)) by Dickson et al. (2021) [50]. Campillo et al. (2021) [32] further developed the analysis to make future predictions of drugs required based on past patterns of drug taking. Consistent treatment behaviour to consecutive rounds of MDA was noted to decrease parasite prevalence.

Aside from demographic factors, perceptions of both the MDA activity and the CDD delivering the treatments were also analysed in some studies. Common themes included awareness of the targeted disease and the role of MDA, perceived individual susceptibility to the target disease, CDD dedication, health benefits, and adverse events related to drug ingestion. Prior knowledge of MDA scheduling was noted to have a significant effect on compliance in two studies. Asfaw *et al*. (2021) [58] noted a 69% lower compliance to MDA, between prior awareness of date versus date and location of MDA activity (AOR 0.31 (95% CI: 0.12–0.82)), whilst Krentel *et al*. (2016) [55] noted compliance increasing with prior awareness of MDA activity (AOR 2.59 (95% CI: 1.0–6.7)). The perception of drugs to be beneficial to participants was also noted to be significantly associated with increased compliance; (AOR 7.33 (95% CI: 4.13–13.02)) [34], (AOR 5.25 (95% CI: 2.55–10.82)) [53], and (AOR 10.74 (95% CI: 5.1–22.6)) [55]. The perceived dedication and attitude of CDDs, as well as the protocols of height measurement were analysed by some authors, proving to be significant factors in increasing participant compliance. Travel time to the MDA distribution site was only collected by one study, delineating an individual’s will to take MDA, with the practicality of doing so (AOR 0.22 (95% CI: 0.14–0.35) >60 minutes, AOR 0.08 (95% CI: 0.04–0.17) 30–60 minutes), reference: < 30 minutes) [58]. Participant comprehension of parasitic disease (AOR 13.68 (95% CI: 1.65–113.57)) [59], or the purpose of MDA (AOR 2.27 (95% CI: 1.44–3.59)) [58] was noted to be significant. Similarly, internal cues to action such as the assumption of own infection status, susceptibility of disease and fear of disease was shown to be significant (AOR 3.628 (95% CI: 1.96–6.73)) [60]. It must be noted however, that despite these large AOR presented throughout the literature, the associated confidence intervals are very wide, reducing the reliability of this interpretation.

## Discussion

This review highlights the paucity of individual longitudinal compliance studies available in the literature for the five (PC)-NTDs. The review aimed to compare reported individual longitudinal compliance recorded by CDDs, referred to as DOT. Despite publications dating back to 2004 [61], and more recently by Shuford *et al*. (2016), detailing the need for increased individual longitudinal compliance monitoring and reporting in the body of NTD research, a single study was identified reporting individual longitudinal compliance recorded by CDDs [17]. The lack of publications fulfilling the review criteria resulted in the inclusion of all papers reporting compliance. As such, the review includes the analyses of very different types of compliance studies, both individual and population-level data (repeated cross-sectional studies), analysing information both recorded by CDDs and self-reported by participants.

The confidence in which these varying measures of compliance can be accepted should be interpreted with care. As previously mentioned, the gold standard to measure individual longitudinal compliance is DOT. This method avoids recall and desirability bias which may impact the compliance figures self-reported by participants when asked if they have ‘ever’ been treated across a defined or undefined time-period. Furthermore, participant self-reporting may also confuse different MDA activities associated with different NTDs, as the few chemotherapeutics available for (PC)-NTD MDA are often provided by overlapping treatment programmes as NTDs are commonly found as co-endemicities. However, despite being the gold standard, it is important to note that DOT may also be subject to recording errors, since CDDs may be under pressure to report high figures. Furthermore, the dataset used to populate compliance calculations should also be critically appraised. If a large proportion of the community is absent from the MDA survey (numerator) and this population was also absent from the baseline survey (the denominator), this will artificially inflate the population assumed to have taken treatment. For example, young male adults undertaking work away from the home setting may miss both the census and MDA activity by virtue of their occupation. The impact this ‘absent population’ may have on control will vary, dependent on the demographic of the population, the transmission intensity in a given setting, and parasite in question. For example, if the same young men were absent from albendazole MDA, this will have a higher risk of hookworm (one of the STH species) or OV morbidity due to the age-dependent distributions of prevalence typically observed for these parasites where most infection occurs in adults not children [62,63].

Even if compliance was to reach 100%, the efficacy of the selected drug against each species should also be noted. For instance, albendazole is distributed for STH control, with different efficacies for each species, ranging from *Ascaris lumbricoides* (91.4%), to *Trichuris trichiura* (50.0%) [6]. Furthermore, repeated MDA rounds at low efficacies or compliance levels may enable the development of drug resistance to the limited number of currently available chemotherapeutics [64]. The donation of these drugs over the past few decades has depended on pharmaceutical companies that manufacture them. At present many of these donation pledges terminate in the mid-2020s [64]. This underscores the importance of reaching high compliance levels in treated communities while the donations continue, not only to reduce the infectious material shed into the environment to reach transmission interruption, but to do so quickly before the development of drug resistance threatens control efforts, or pharmaceutical companies reduce their current donation pledges.

Currently, there is much heterogeneity in the terminologies employed to define coverage and compliance including the species-specific guidelines provided by WHO. For example, in the case of LF control, two different denominators are defined for the coverage parameter in the same guideline. Drug coverage is defined as the proportion of the *eligible* (also referred to as targeted) population that *swallow* drugs. However, the programmatic drug coverage is defined as the proportion of the *total* population swallowing drugs (recommended to reach 80%). Drug coverage surveys are also suggested whereby a sub-sample of the population is questioned on their treatment behaviour, including coverage and compliance treatment behaviours [65]. This is an early example of the recommendation of detailed recording of compliance in NTD research. As such, the focus on LF compliance by WHO (defined as coverage in the guideline, but referred to as compliance in this review), is reflected in the number of reviewed studies, as those reporting compliance for LF control were most numerate.

In contrast, the guidance provided for STH control defines coverage as the proportion of the target population *reached* [66]. Systematic non-treatment (referred to as systematic non-compliance) is mentioned in the context of LF control, defined as the individuals who never ingest the medicines in any MDA round, but is absent from the analogous STH control guidelines. It would be beneficial for future editions of the WHO roadmap, and species-specific control guidelines, to clearly differentiate between coverage and compliance parameters, as current terminology use are not uniform across infections. Common definitions need to be adopted by WHO across all NTD infections. The mismatch of parameter definitions currently provided by WHO is particularly problematic for the species recently targeted for integrated control, as these will need to be adhered to across research groups and programmes for synchronous and efficient monitoring and evaluation. The continued heterogeneity in MDA parameter definitions in NTD research highlighted in this review pertains despite the call for consistency made in the Shuford et al 2016 review of the same issue. Regardless of the parameter used by different studies and programmes, the terminology and assumptions made in the calculation of compliance should be clearly defined within the methods section of publications to enable the scientific audience to interpret the data unambiguously. It is hoped that future WHO guidelines will align on these parameters and associated terminology, to set standards for clear definitions for the NTD research community.

This review reports a large difference between coverage (receiving treatment) to compliance (swallowing treatment) when comparing the data reported by studies, mirroring the 22% coverage-compliance gap previously published by Babu *et al*. (2014) [5] and the 12.1% gap reported by Sitikantha *et al*. (2019) [67]. In practice, this highlights the fact that a significant proportion of individuals that are un-treated are considered within the parameter of coverage. Programmes targeted with reaching coverage-based targets that conflate this parameter with compliance could result in the premature cessation of programmes as a higher proportion of the community are assumed to be treated than in reality [55]. For example, if coverage reaches a defined target e.g. 75% of a population, and compliance is only 80%, then 750 will have received treatment, but only 600 will have swallowed treatment. As such, beyond the clear definition of the parameters used, a stipulation from WHO to routinely report compliance alongside coverage would be beneficial in improving the accuracy of reporting of individuals that have actually been effectively treated. Reflecting the lack of MDA compliance guidance from WHO, the published peer reviewed literature on longitudinal monitoring of compliance across the NTD research landscape was found to be very limited. Fewer studies still reported age- and gender-stratified demographic factors influencing treatment behaviour. The collection of such data would facilitate the analysis of trends in MDA behaviour, therefore enhancing future MDA rounds through targeted sensitisation activities to improve compliance.

The importance of monitoring compliance at the individual level has been noted in past publications [68], and the impact of longitudinal compliance patterns upon control programme outcome has been extensively analysed, employing mathematical models of parasite transmission and MDA impact. For example, Hardwick *et al*. (2021) created a framework of compliance (referred to as ‘adherence’), proposing the parameter *ω*_*n*_ to measure the strength of association of previous individual compliance behaviour at MDA with that of a future round of MDA using stochastic individual-based simulations [15]. This uses the notion of conditional probability, whereby the behaviour of an individual at one round of MDA is not independent of their behaviour at a previous round (systematic behaviour). Applying this framework to the age and gender-stratified compliance dataset collected by TUMIKIA across four biannual rounds of MDA, Hardwick et al showed that despite high coverage figures, past behaviour-dependent compliance (or non-compliance) lowered the potential of hookworm elimination by 43% and 23% in the two surveyed communities, respectively, compared to predictions based upon random compliance (behaviour at one round independent of behaviour at previous round). Past behaviour-dependent compliance was particularly apparent in males aged >30 years old. This conditional probability model demonstrates the impact that not only compliance, but also demographic factors, have upon reaching the goal of transmission elimination. Of the 89 studies included in this review, compliance stratified by location, gender, and age were rarely recorded. The importance of these co-variates is clear in many studies. Presenting compliance data at the population level will potentially aggregate important behavioural heterogeneities within communities which need programmatic attention. Future WHO guidelines to Ministries of Health would benefit from not only including coverage stratifications by age and gender, but to reflect this in compliance reporting. MDA behavioural treatment patterns greatly impact MDA success, and concomitantly this will further impact the number of required rounds of MDA, and the magnitude of targeted coverage taking account of compliance behaviour, to achieve control programme goals.

Currently, there are two longitudinal studies underway that are recording individual longitudinal compliance patterns to MDA for STH control. These are the Geshiyaro [69] and DeWorm3 projects [70]. These studies employ the recommended MDA monitoring strategies presented here and will provide demographically stratified individual longitudinal compliance data. Of most importance in these studies, is the inclusion of individual longitudinal compliance recorded by DOT. This is considered the gold standard of reporting in other disease fields that routinely follows longitudinal compliance such as, for example, that of HIV treatment [71] and angiotensin-converting-enzyme (ACE) inhibitors for blood pressure control [72]. Lessons learnt from other areas of public health are of high relevance to NTD control programmes.

NTD research is sometimes hindered by small budgets and limited study duration, thus the high cost of the reporting and measurement infrastructure necessary for accurately monitoring individuals over several time points to capture longitudinal compliance may not be realistic for most NTD control programmes. This is reflected in the proportion of longitudinally recorded compliance studies (n = 3) versus cross-sectional surveys using participant recollection (n = 22) documented in this review. As such, cross-sectional surveys conducted on a sub-sample of the treated population who are asked to recall if they have ever been treated, or preferably, how many treatment rounds they have participated in, is more realistic for most NTD control programmes in resource poor settings. Despite the decrease in accuracy of subject recall compared to DOT recorded by CDDs (which is also subject to its own biases), a cross-sectional snapshot of compliance in the population will provide a complementary measure to coverage and will help elucidate the impact of an MDA activity upon the local parasite burden. Furthermore, if such programmes fail to reach targeted prevalence and intensity levels, compliance data can accordingly be targeted to the demographic groups with low compliance patterns.

## Conclusions

There is a paucity of individual longitudinal compliance monitoring of MDA treatment for the control of (PC)-NTDs. This review has shown that there has been a small increase in the number of papers published which contain clearly defined compliance measurements, from a very low baseline in 2016. It must be noted, however, that the individual monitoring of compliance is demanding in terms of cost and time since it requires longitudinal follow-up of individuals including detailed information on local demography to ensure the correct denominators are employed in calculating compliance. In addition to the enhanced accuracy of coverage and compliance recording required, a greater focus is needed in recording population size and age structure. A census or register of all participants must first be taken to monitor MDA behavioural patterns, and due note taken of births, deaths and migration if the study population is followed over many years. As a result, prospective cross-sectional studies interviewing participants on their treatment history at one point in time were most numerate in the reviewed literature. This is not the gold standard method of longitudinal compliance measurement, but the information that a control programme or local ministry of health will gain, such as the definition of systematic or random compliance patterns in defined demographic sub-groups, will be crucial for guiding future MDA rounds to reach the goals of reducing infection to very low levels, or indeed eliminating transmission in defined populations or regions. Greater attention by WHO is required to both unify definitions on both coverage and compliance, and to provide more detailed guidance on their measurement for the five (PC)-NTDs.

## Supporting information

S1 PRISMA Checklist PRISMA guidelines for full text of review and abstract. (DOCX)

S1 Note The search strategy employed to generate the review. (DOCX)

S1 Data Workflow of screening and inclusion of studies as defined by the PRISMA guidelines. (DOCX)

S2 Data The complete 89 studies identified as containing compliance data. (XLSX)
