# Supplementary material for: Individual longitudinal compliance to neglected tropical disease mass drug administration programmes, a systematic review
Source: PLoS Negl Trop Dis. 2023 Jul 17;17(7):e0010853. doi: 10.1371/journal.pntd.0010853 (PMC10374057; doi:10.1371/journal.pntd.0010853)
Supplement: S1 Note — (DOCX) [file pntd.0010853.s002.docx]

# Search strategy

The following search strategy was employed for the review, allowing for minor syntax changes between search engines.

*"soil-transmitted helminths" OR helminth* OR Helminthiasis OR ascari* OR hookworm OR trichur* OR strongyl* OR schistosom* bilharz* OR onchocerc* OR "river blindness" OR "elephantiasis" OR "lymphatic filariasis" OR "Wuchereria bancrofti" OR "Brugia malayi" OR wucherer* OR brugia* OR ancylostomatoidea OR Ancylostoma OR Necator OR trachoma*) AND ("compliance" OR "non-compliance" OR "adherence" OR "non-adherence" OR "refusal" OR "medication adherence" OR "systematic non-treatment" OR "systematic treatment" OR "systematic adherence" OR "systematic compliance" OR "never treated" OR "directly observed treatment" OR "mass drug administration" OR "systematic non-compliance" OR "coverage" OR "treatment coverage" OR "surveyed coverage" OR "uptake" OR "participation"*

Confined to studies:

- Published from 01/01/2016 – 01/01/2022
- Written in English or Spanish
