# Supplementary material for: Individual longitudinal compliance to neglected tropical disease mass drug administration programmes, a systematic review
Source: PLoS Negl Trop Dis. 2023 Jul 17;17(7):e0010853. doi: 10.1371/journal.pntd.0010853 (PMC10374057; doi:10.1371/journal.pntd.0010853)
Supplement: S1 Data — (DOCX) [file pntd.0010853.s003.docx]

Records screened* (n = 2529)

Total studies included in review (n = 89):

Longitudinal compliance (57)

Cross-sectional compliance (n = 32)

Studies assessed for eligibility (n = 159)

Studies identified from reference search of included studies (n = 1)

Studies excluded (n = 70):

Irrelevant (n = 40)

Coverage studies (n = 30)

Studies sought for retrieval (n = 164)

Studies not retrieved (n = 6)

Reports excluded (n = 2365):

Qualitative (n = 40)

Modelling (n = 45)

Irrelevant (n = 2280)

Records identified (n = 3649):

(PubMed, Web of Science)

**Identification of studies from databases**

*categorisation of studies was completed using the assistance of artificial intelligence software (AI), Ryann. All decisions were manually confirmed by at least two authors.
